# Isolation of an Equine Foamy Virus and Sero-Epidemiology of the Viral Infection in Horses in Japan

**DOI:** 10.3390/v11070613

**Published:** 2019-07-05

**Authors:** Rikio Kirisawa, Yuko Toishi, Hiromitsu Hashimoto, Nobuo Tsunoda

**Affiliations:** 1 Laboratory of Veterinary Virology, Department of Pathobiology, School of Veterinary Medicine, Rakuno Gakuen University, Ebetsu, Hokkaido 069-8501, Japan; 2 Shadai Stallion Station, Abira-cho, Hokkaido 059-1432, Japan; 3 Shiraoi Farm, Shiraoi-cho, Hokkaido 059-0901, Japan

**Keywords:** equine foamy virus, isolation, Japan, sero-epidemiology, spumaretrovirus

## Abstract

An equine foamy virus (EFV) was isolated for the first time in Japan from peripheral blood mononuclear cells of a broodmare that showed wobbler syndrome after surgery for intestinal volvulus and the isolate was designated as EFVeca_LM. Complete nucleotide sequences of EFVeca_LM were determined. Nucleotide sequence analysis of the long terminal repeat (LTR) region, *gag, pol, env, tas,* and *bel2* genes revealed that EFVeca_LM and the EFV reference strain had 97.2% to 99.1% identities. For a sero-epidemiological survey, indirect immunofluorescent antibody tests were carried out using EFVeca_LM-infected cells as an antigen against 166 sera of horses in five farms collected in 2001 to 2002 and 293 sera of horses in eight farms collected in 2014 to 2016 in Hokkaido, Japan. All of the farms had EFV antibody-positive horses, and average positive rates were 24.6% in sera obtained in 2001 to 2002 and 25.6% in sera obtained in 2014 to 2016 from broodmare farms. The positive rate in a stallion farm (Farm A) in 2002 was 10.7%, and the positive rates in two stallion farms, Farms A and B, in 2015 were 40.9% and 13.3%, respectively. The results suggested that EFV infection is maintained widely in horses in Japan.

## 1. Introduction

Foamy viruses (FVs) belong to the subfamily *Spumaretrovirinae* within the family *Retroviridae* [1]. FVs have been isolated from a wide range of mammals, including nonhuman primates [2,3,4,5], cats [6], cows [7], horses [8] and bats [9], and it has been shown that they establish lifelong infection [10,11]. FV infections have not been shown to be associated with any defined disease [1,12]. The non-pathogenicity of FVs is an essential factor for the development of a foamy viral vector in gene therapy [13]. The FV genome consists of genes encoding canonical retroviral Gag, Pol and Env proteins and a regulatory protein Tas and an accessory protein Bet [1].

The prevalence of simian FVs has been studied in detail, but there have been few studies on the prevalence of other animal FVs [14]. The prevalence of feline FV (FFV) in domestic cats and wild cats was reported to range from about 30% to 100% depending on sex, age, and the geographical region [14,15,16,17,18,19,20,21,22]. The prevalence of bovine FV (BFV) infection in cattle was reported to range from 7% to 45% [23,24,25,26]. The prevalence of equine FV (EFV) in horses has not been reported.

In 2000, equine foamy virus was isolated for the first time from blood samples of naturally infected healthy horses after co-cultivation of phytohemagglutinin (PHA)-activated lymphocytes derived from sero-positive horses with permissive human U373-MG cells and hamster BHK21 cells [8]. Nucleotide sequence analysis revealed that EFV is phylogenetically close to non-primate FVs, especially BFV. There has been no further isolation of EFV since the first isolation in 2000.

In this report, the first isolation of EFV in Japan (the second isolation of EFV in the world) in primary horse kidney cells co-cultured with fresh peripheral blood mononuclear cells (PBMC) from a broodmare showing wobbler syndrome after surgery for intestinal volvulus and the molecular characterization of the isolated virus are described. The results of a serological survey using the Japanese EFV isolate in thoroughbred horses in Japan are also described.

## 2. Materials and Methods

### 2.1. Cell Cultures and Virus Isolation

Primary horse fetal kidney (HFK) cells were prepared according to the standard method from a fetal kidney that was obtained from a euthanized pregnant mare due to the judgment of a poor prognosis for a forelimb fracture, and the cells were cultured in MEM supplemented with 10% fetal calf serum (FCS) as the growth medium at 37 °C. A blood sample from a horse (Horse A) that exhibited wobbler syndrome the day after a surgical operation for intestinal volvulus in an equine hospital, not in our medical center of Rakuno Gakuen University, as veterinary medicine was collected in heparin-containing tubes on October 1, 2001, and peripheral blood mononuclear cells (PBMC) were isolated by Ficoll-Paque gradients (density of 1.077 g/mL). The PBMC were co-cultured with HFK cells in culture dishes (35 mm in diameter) in the growth medium for virus isolation at 37 °C under a 5% CO_2_ atmosphere. The culture medium was removed the next day, fresh MEM supplemented with 4% FCS as a maintenance medium was added, and the cells were cultured at 37 °C. The cultured cells were observed daily and the maintenance medium was changed at 4-day intervals until the appearance of a cytopathic effect (CPE). A CPE was observed 10 days after the start of cultivation and the HFK cells showing a CPE were detached by trypsin-EDTA solution and harvested as virus-infected single cells 4 days after the appearance of a CPE. The infected cells were stored at −80 °C using CELLBANKER I (Takara Bio Inc., Kusatsu, Shiga, Japan) as the cryopreservation medium. Serum of Horse A was collected on October 1, 2001. Horse A was euthanized due to the judgment of a poor prognosis about 1 week after the operation. We also used conserved sera of Horse A that had been stocked monthly in our laboratory from January 2000.

### 2.2. DNA Extraction

Total DNA was extracted from HFK cells showing a CPE (about 80% of the cells) as described previously [27].

### 2.3. Polymerase Chain Reaction (PCR) for Detection of the Equine Foamy Virus Genome

To detect EFV DNA in cells showing a CPE, PCR assays targeting LTR were carried out using the Expand High Fidelity PCR system (Roche Diagnostic GmbH, Mannheim, Germany) and primers listed in Table 1, which were designed on the basis of the complete EFV sequence (GenBank AF201902) by using DNASIS Pro (Hitachi Software Engineering Co., Ltd., Tokyo, Japan). PCR amplification was carried out as described previously [28] under the following conditions: an initial denaturation step of 94 °C for 5 min, 35 cycles of 94 °C for 30 s, 55 °C for 30 s, 72 °C for 1 min and 30 s, and a final extension step of 72 °C for 5 min. The PCR products were purified and sequenced as described below.

### 2.4. Restriction Enzyme Digestion and Southern Blot Hybridization

The DNA extracted from cells showing a CPE was digested to completion with restriction endonuclease *Bam*HI under conditions recommended by the manufacturer (Takara Bio Inc., Tokyo, Japan). The digested fragments were separated by electrophoresis in 0.7% agarose gels in Tris-acetate-EDTA buffer (40 mM Tris-acetate, 1 mM EDTA, pH 8.0) and were transferred to nitrocellulose filters (0.45 μm, Schleicher & Schuell, Dassel, Germany) according to the method by Southern [29]. The filters were pre-hybridized for 2 h and then hybridized for 14 h with a probe of the isolated viral LTR labeled with the non-isotopic reagent digoxigenin-dUTP [27]. An enzyme immunoassay kit (Roche Diagnostics, Basel, Switzerland) was used for detecting hybridized fragments. For a DNA molecular weight marker, *Hin*dIII-digested lambda DNA labeled with digoxigenin-dUTP (Roche Diagnostics) was used.

### 2.5. Sequence and Phylogenetic Analyses

Regions coding for Gag, Pol, Env, Tas and Bet were amplified by PCR with the Expand High Fidelity PCR system (Roche Diagnostics) and each of the specific primers listed in Table 1 and Table 2. The PCR products were purified by Chroma spin columns (Clontech Laboratories, Inc., Mountain View, CA, USA) or a High Pure PCR Product Purification kit (Roche Diagnostic) and used for sequencing. Sequencing was conducted in Hokkaido System Science Co. Ltd. (Sapporo, Japan) using specific primers and walking primers. Sequence analyses were conducted by DNASIS Pro (Hitachi Software Engineering Co., Ltd., Tokyo, Japan). Phylogenetic analysis of the nucleotide sequences was conducted by using MEGA7 software with 1000 bootstrap replicates of the neighbor-joining method [30]. Evolutionary distances were estimated according to the Kimura 2-parameter method [31]. The DDBJ accession number assigned to the complete sequence of the analyzed isolate is LC381046.

### 2.6. Serum Samples

Sera obtained from horses in 10 farms (Farms A to J) in Hokkaido in Japan were used for a sero-epidemiological survey. Farms A and B were stud farms, and the others were breeding farms. In 2001 to 2002, sera were collected from 28 stallions in Farm A on June 7, 2002; 72 mares in Farm C on June 13, 2002; 25 mares in Farm D on April 17, 2001; and 29 mares in Farm E and 12 mares in Farm F on June 30, 2002. In 2014 to 2016, sera were collected from 44 stallions in Farm A on June 30, 2015; 15 stallions in Farm B on May 15, 2015; 107 mares in Farm C on June 30, 2015; 25 mares in Farm D on January 26, 2015; 30 mares in Farm G on March 11, 2015; 39 mares in Farm H on January 17, 2014; 22 mares in Farm I on January 28, 2016; and 11 mares in Farm J on October 31, 2016. For the broodmare from which EFV was isolated (Horse A, Farm C), serum collected on October 1, 2001 (date of onset of wobbler syndrome) and sera collected on February 14, 2001; October 11, 2000; and January 26, 2000 were used. All of the serum samples were initially sent to our laboratory to test for equine herpesvirus 1 (EHV-1) infection. The serum separation procedure was as follows. A blood sample from each horse was collected in a plain tube and was allowed to clot by leaving it at room temperature. The clot was removed by centrifugation at 1000× g for 10 min and the resulting supernatant, designated as a serum, was transferred to a clean polypropylene micro tube. After inactivation of the complement at 56 °C for 30 min, the serum was used for a serological test of EHV-1 infection. After the test, the serum was stored at −20 °C.

### 2.7. Indirect Immunofluorescence Assay (IFA) for Detection of Antibodies to EFV

EFV-infected HFK cells were detached by trypsin-EDTA solution and washed three times with phosphate-buffered saline (PBS, pH 7.4) by centrifugation at 200× *g* for 5 min. The cells were re-suspended in a small volume in PBS and smeared on a 15-well multitest slide glass (MP Biomedicals, LLC, Solon, OH, USA) and then fixed in 100% acetone on ice for 30 min. Equine sera were diluted serially from 1:20 to 320 and incubated with the fixed cells at 37 °C for 30 min in an incubation chamber. The cells were washed three times with PBS. After drying the cells at room temperature, the cells were incubated with 1:80 diluted fluorescein isothiocyanate conjugated goat-anti horse IgG (Jackson Immuno Research Inc., West Grove, PA, USA) at 37 °C for 30 min. After a final wash, infected cells were visualized under a fluorescence microscope (Olympus, Tokyo, Japan). An IFA titer of 20 or greater was regarded as positive. Uninfected HFK cells were used as control cells.

## 3. Results

### 3.1. Virus Isolation and Identification

**Figure 1:**
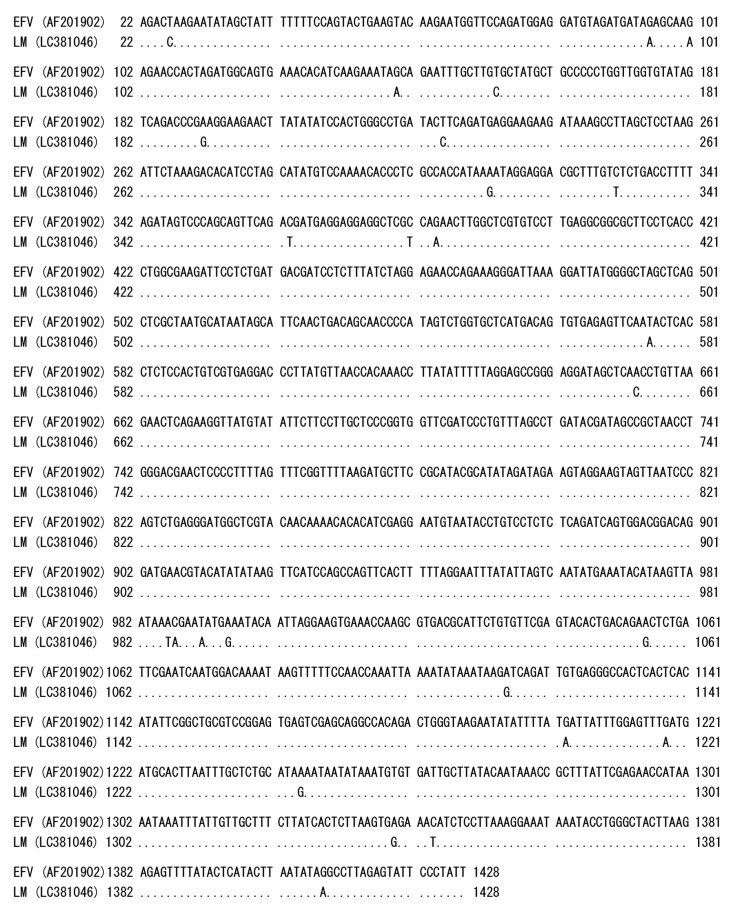
Comparison of the nucleotide sequences of LTR regions in the Japanese isolate EFVeca_LM and reference EFV. Identical nucleotides are indicated by dots. Numbers on the left and right sides are the nucleotide positions of EFV complete genome sequence (AF201902).

**Figure 2:**
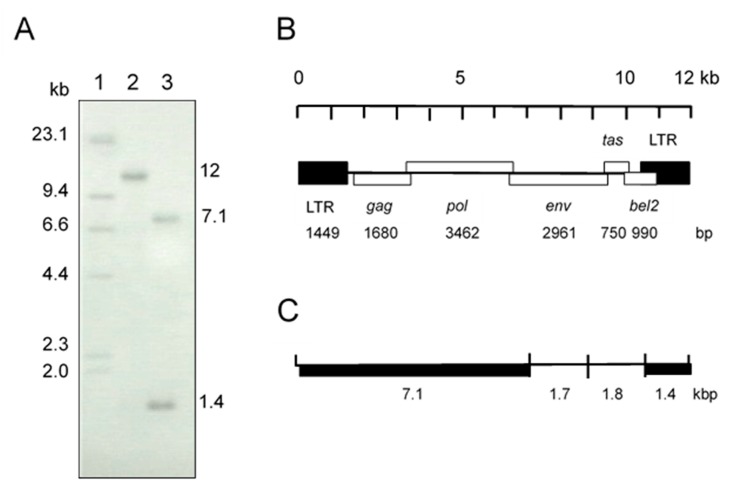
(**A**) Southern blot analysis of total DNA from HFK cells infected with the isolate. The LTR region was used as a probe. Lane 1: molecular weight marker, lambda DNA *Hin*dIII digest labeled with digoxigenin-dUTP, Lane 2: uncut DNA, Lane 3: *Bam*HI-digested DNA. (**B**) EFV genomic structure. (**C**) *Bam*HI restriction map of the EFV genome. Fragments of thick lines were detected by the LTR probe in lane 3 in (**A**).

Initially, we suspected a neurological type of equine herpesvirus 1 infection in a horse showing clinical symptoms of wobbler syndrome. However, co-cultivation of PBMC from the affected horse and HFK cells did not show any herpes viral-like CPE after incubation for 1 week. At 10 days after cultivation, small syncytia were observed and they gradually increased in size, and many vacuoles were observed in the syncytia. These morphological changes resembled the foamy CPE in response to FV infection [1]. The isolate was designated as EFVeca_LM. EFVeca_LM was highly cell-associated in HFK cells, and cell-free virus was not released in culture supernatants. Cell-free virus was also not obtained after three cycles of freeze-and-thawing of EFVeca_LM-infected HFK cells. By PCR amplification using primers for the LTR region of EFV, identical products with the same estimated size, approximately 1450 bp, were observed in agarose gel electrophoresis. The nucleotide sequence identity of the amplified products without primer sequences was 98.2% (1381/1407) against that of EFV (Figure 1). In Southern blot analysis, the LTR probe detected a 12-kbp fragment in DNAs from virus-infected cells without restriction enzyme digestion and 7.1-kbp and 1.4-kbp fragments in *Bam*HI-digested DNAs (Figure 2A). By analogy to the EFV genome, the 12-kbp fragment represents unintegrated linear viral genomic DNA and the 7.1-kbp and 1.4-kbp fragments correspond to about 60% of the region of the genome from the 5’ end and the LTR region located at the 3’ end, respectively (Figure 2B,C). We could not detect the integrated viral DNA in host chromosomal DNA in Figure 2A. Possible reasons were that copy numbers of the integrated DNA were extremely low compared to those of unintegrated viral DNA and that sensitivity of our Southern blot analysis was insufficient for detecting the integrated viral DNA. The same results were obtained by Tobaly-Tapiero et al. [8].

Altogether, the isolated virus, EFVeca_LM, was identified as equine foamy virus belonging to the *Spumaretrovirinae* subfamily.

EFV IFA titers of Horse A are shown in Table 3. EFV antibody already existed in the serum collected at the time of onset of wobbler syndrome. In three conserved sera, EFV antibodies were also detected and showed almost the same IFA titers as that in serum collected at the time of onset of wobbler syndrome. The titers of all of the tested sera against uninfected control HFK cells were less than 20.

### 3.2. Sequence Analysis

The provirus DNA of EFVeca_LM was completely sequenced and found to be 12,034 bp. The complete provirus genomic sequence of EFVeca_LM was submitted to DDBJ under the accession number LC381046. The complete genomic sequences of EFVeca_LM were compared to those of EFV (GenBank AF201902) (Table 4). The LTR nucleotide sequences of EFVeca_LM and EFV showed 98.2% identity. The nucleotide sequence and amino acid sequence of the *gag* gene showed 98.6% and 99.1% identities, respectively. The nucleotide sequence and amino acid sequence of the *pol* gene showed 98.6% and 99.1% identities, respectively. The nucleotide sequence and amino acid sequence of the *env* gene showed 98.3% and 98.5% identities, respectively. The nucleotide sequence and amino acid sequence of the *tas* gene showed 99.1% and 100.0% identities, respectively. The nucleotide sequence and amino acid sequence of the *bel2* gene showed 97.2% and 97.3% identities, respectively.

**Figure 3:**
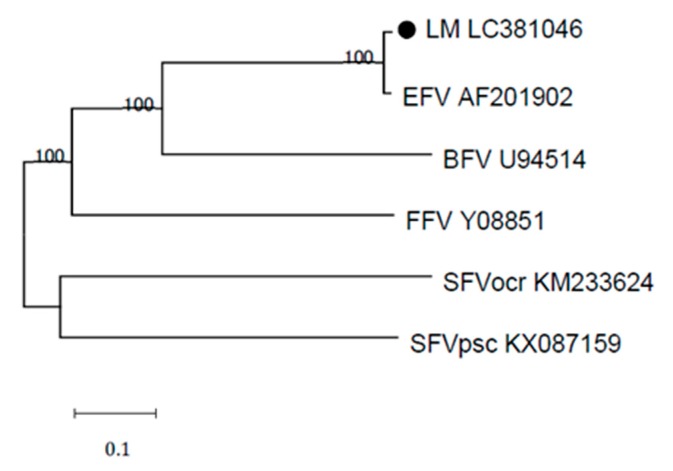
Phylogenetic analysis of the Japanese isolate EFVeca_LM (LM LC301046) and other foamy virus strains. The phylogenetic tree was generated using complete nucleotide genome sequences. EFVeca_LM is indicated by a closed circle. Bootstrap values less than 50% are not shown on the corresponding nodes. BFV: bovine foamy virus, FFV: feline foamy virus, SFVocr: simian foamy virus Otolemur crassicaudatus, SFVpsc: simian foamy virus Pan troglodytes schweinfurthii.

Phylogenetic comparisons of the full-length provirus genome of EFVeca_LM and those of various animal foamy virus isolates revealed that EFVeca_LM belonged to the same clade as EFV (Figure 3).

### 3.3. Sero-Epizootiology

To examine the prevalence of EFV antibodies in Japanese horses, we conducted IFA tests using EFVeca_LM and a total of 166 sera obtained from one stallion farm (Farm A) and four broodmare farms (Farms C to F) in 2001 to 2002 and a total of 293 sera obtained from two stallion farms (Farms A and B) and six broodmare farms (Farms C, D, G to J) in 2014 to 2016 (Table 5). The titers of all of the tested sera against uninfected control HFK cells were less than 20. All of the farms had EFV antibody-positive horses. The positive rates in sera obtained from broodmare farms in 2001 to 2002 ranged from 20.7% to 28.0% (average: 24.6%), and the positive rates in sera obtained from broodmare farms in 2014 to 2016 ranged from 12.8% to 35.5% (average: 25.6%). The positive rate in sera obtained from a stallion farm (Farm A) in 2002 was 10.7% and the positive rates in sera obtained from two stallion farms, Farm A and Farm B, in 2015, were 40.9% and 13.3%, respectively. Average ages of broodmares for which sera were tested and broodmares for which sera were antibody-positive in 2001 to 2002 were 9.8 years and 10.3 years, respectively. The average ages of broodmares for which sera were tested and broodmares for which sera were antibody-positive in 2014 to 2016 were 10.7 years and 12.0 years, respectively. The average ages of stallions in Farm A for which sera were tested in 2002 and in 2015 were 9.1 years and 11.3 years, respectively. The average ages of stallions in Farm A for which sera were antibody-positive in 2002 and in 2015 were 8.0 years and 13.7 years, respectively. Positive rates in sera obtained from Farms A and C in 2015 were higher than those in sera obtained in 2002, and there was a relationship between the positive rate and aging in Farms A and C in 2015 (Table 6). In Farm A, the positive rate in sera obtained in 2015 from stallions aged 15 to 24 years (83.3%) was clearly higher than the positive rates for other age groups (27.3% in stallions aged 4 to 9 years and 20.0% in stallions aged 10 to 14 years). In Farm C, the positive rates in sera obtained in 2015 from broodmares aged 10 to 14 years old (42.0%) and broodmares aged 15 to 24 years (56.3%) were clearly higher than the positive rate for broodmares aged 4 to 9 years (19.5%). In Farm C in 2002, the positive rates in sera were similar for the three age groups. The average ages of horses reared in Farms A and C had increased from 9.1 years to 11.3 years and from 9.4 years to 10.6 years in 2015, respectively. In Farm A, the same four horses were reared both in 2002 and 2015, and two horses were EFV antibody-positive in 2002 (one 4-year-old horse and one 10-year-old horse) and also antibody-positive in 2015 (one 17-year-old horse and one 23-year-old horse). The remaining two horses did not possess EFV antibody in 2002 (one 9-year-old horse and one 10-year-old horse) but possessed EFV antibody in 2015. In Farm C, there was no same horse reared both in 2002 and 2015.

## 4. Discussion

In this study, we isolated an EFV strain for the first time in Japan from PBMC obtained from a horse that showed symptoms of wobbler syndrome after surgery for intestinal volvulus. In general, spumaretroviruses have no pathogenicity in animals. Although pathogenicity of EFV has not been clearly demonstrated, the EFV we isolated and the symptoms observed in the horse might have no relationship. Concerning EFV isolation from PBMC, Tobaly-Tapiero et al. [8,32] reported that it took 4 weeks to isolate EFV in a highly FV-permissive adherent cell line (either human U373-MG cells or hamster BHK21 cells) after pre-cultivation of PBMC with the mitogenic lectin PHA-P for 2 days. On the other hand, we were able to isolate EFV from mitogen-untreated PBMC in HFK after co-cultivation for 10 days. Recently, we isolated another EFV from PBMC obtained from an EFV antibody-positive horse in HFK after co-cultivation for 10 days. These results suggested that isolation of EFV from PBMC could be conducted by co-cultivation with HFK without pre-cultivation of PBMC with mitogens. However, in our system, cell-free EFV was not released into the supernatant of EFV-infected HFK cell culture. Furthermore, cell-free EFV was not produced after three cycles of freezing and thawing of infected cells in culture medium. BFV is also highly cell-associated and spreads mainly through cell-to-cell transmission [33,34,35,36]. Most primate foamy viruses budded from intracellular membranes and cell-free viruses were produced following three cycles of freezing and thawing of infected cells [32]. However, Tobaly-Tapiero et al. [32] obtained cell-free EFV in the culture supernatant of EFV-infected human U373-MG cells without a freezing and thawing procedure. In ref. [32], it was reported that this phenomenon might be due to the lack of a dilysine motif in the C-terminus of the primate Env glycoprotein [37,38]. Our EFV isolate also lacked the dilysine motif. Therefore, the process of EFV replication in cells might be different depending on the cell type. We plan to propagate our EHV isolate in human U373-MG cells to confirm the results obtained by Tobaly-Tapiero et al. [32].

Nucleotide sequence data for EFVeca_LM showed high identities to those for prototype EFV (97.2% to 99.1% in various coding regions). The FFV clade clusters with a sequence identity of about 94% to 99% in partial *gag* and *pol* genes [14,39]. However, in the partial *env* gene, FFVs were divided into two distinct genotypes corresponding to two distinct serotypes [39,40,41,42]. In BFVs, phylogenetic analysis of complete genomic sequences revealed two clades, the European clade and non-European clade [39]. A recent Japanese BFV isolate belonged to the non-European clade based on results of phylogenetic analysis of partial *env* gene sequences [36]. Therefore, EFV might also be divided into two or more clades if more EFV isolates are obtained worldwide.

Since sero-prevalence of the EFV antibody in broodmares in Japan was about 25% in both the periods 2001 to 2002 and 2014 and 2016, it is thought that EFV infection might persist in almost a constant percentage of horse populations. This is the first report on sero-epidemiology in horses, though preliminary studies in horses in Poland showed the presence of provirus nucleic acid of EFV in about 15% of the tested animals [43]. In our study, the EFV-positive rate increased in an age-dependent manner, especially in Farms A and C in 2015. Furthermore, the average ages of horses reared in both farms in 2015 were slightly increased compared to those in 2002. In Farm A, two horses were sero-converted between 2002 and 2015. The mechanism of EFV transmission is not known, but it is likely that the longer the time spent in the same farm, the greater is the chance to become sero-positive [23]. A significant interaction between age and sero-positivity to BFV has also been reported and it was suggested that this phenomenon is due to horizontal transmission [23]. Furthermore, since the EFV-antibody positive rate in stallions of Farm A in 2015 was the highest among all farms in both periods, sexual transmission might have occurred in the breeding season. In any case, it is thought that most infections result from horizontal transmission.

Fortunately, we have sera from Farms A and C that have been stocked monthly for about 18 years in our laboratory, and we plan to conduct a detailed serological survey to determine the epidemiology of EFV infection in horses. Furthermore, we plan to isolate other EFVs from sero-positive horses and examine the molecular epidemiology of EFV infection in horses in Japan.

## Figures and Tables

**Table 1:** Primers used in PCR amplification.

Primer	Sequence (5’–3’)	Location ^1^
LTR-F	TGTCATGGAATGAGGATCCAG	1–21
		10587–10607
LTR-R	ATTGTCGCGGTATCTCCTTAA	1449–1429
		12035–12015
Gag-F	AGATACCGCGACAATTGGCG	1435–1454
Gag-R3	CCATTGTCCCGAGGTAAATC	1637–1618
Gag-F3	AAGAAGAGGCCCTGGAAGAA	3071–3090
Gag-R	AGGGACACAAGTTATTTCAGCTC	3246–3224
Pol-F	GGCGTTATTGAAGGCATTTG	4670–4690
Pol-R	CCCATACCTGCTGAATGTTG	4997–4978
Env-F	ATGACACCTCCAATGACTCTAC	6536–6557
Env-R3	CAGGTATGGCCTCTCTGAT	6617–6599
Env-R	TTATTCTCCTTTGTCCTCTC	9496–9477
Env-F2	TTTGGGTAAAGTACCAGCCTC	8113–8133
Env-R2	GGATAAGTCCACTTCCCAGAG	9528–9508
TAS-F	AGGATATTATCATGGCTAGCA	9431–9451
TAS-R	ATGGTTCTCGAATAAAGCGGT	11885–11865
		1299–1279

^1^ Location at the complete nucleotide sequence of EFV (Genbank AF201902).

**Table 2:** PCR-amplified regions.

Primer Pair	Amplified Region ^1^	PCR Product Size (bp)
LTR-F & LTR-R	1–1429, 10587–12035	1429
LTR-F & Gag-R3	1–1637	1637
Gag-F & Gag-R	1435–3246	1812
Gag-F3 & Pol-R	3071–4997	1927
Pol-F & Env-R3	4670–6617	1948
Env-F & Env-R	6536–9496	2961
Env-F2 & Env-R2	8113–9528	1416
TAS-F & TAS-R	9431–11885	2454

^1^ Region at the complete nucleotide sequence of EFV (Genbank AF201902).

**Table 3:** EFV antibody titers determined by IFA tests in Horse A.

	Serum Collection Date			
	26 Jan, 2000	11 Oct, 2000	14 Feb, 2001	1 Oct, 2001 ^1^
IFA titer	160	160	160	80

^1^ Date of onset of wobbler syndrome.

**Table 4:** Identities of nucleotide sequences and amino acid sequences of an isolated virus and EFV.

	Identity (%)	
Region	Nucleotide Sequence	Amino Acid Sequence
LTR	98.2 (1449) ^1^	
*gag*	98.6 (1680)	99.1 (559) ^2^
*pol*	98.6 (3462)	99.1 (1153)
*env*	98.3 (2961)	98.5 (986)
*tas*	99.1 (750)	100.0 (249)
*bel2*	97.2 (990)	97.3 (329)

^1^ Number in parenthesis is the size of the nucleotide sequence; ^2^ Number in parenthesis is the size of the amino acid sequence.

**Table 5:** Prevalence of EFV antibody in horses in Hokkaido in Japan.

		In 2001 to 2002		In 2014 to 2016	
Farm	Category	Collection Date	Positive/Negative/Tested Sera	Collection Date	Positive/Negative/Tested Sera
A	Stallion	7 Jun, 2002	3 (8.0y) ^1^/ 25 (9.6y)/	30 Jun, 2015	18 (13.7y)/ 26 (9.7y)/
			28 (9.1y) ^2^ (10.7%) ^3^		44 (11.3y) (40.9%)
B	Stallion			15 May, 2015	2 (10.0y)/ 13 (10.8y)/
					15 (10.7y) (13.3%)
C	Broodmare	13 Jun, 2002	18 (9.6y)/ 54 (9.4y)/	30 Jun, 2015	38 (12.0y)/ 69 (9.9y)/
			72 (9.4y) (25.0%)		107 (10.6y) (35.5%)
D	Broodmare	17 Apr, 2001	7 (11.0y)/ 18 (8.6y)/	26 Jan, 2015	7 (11.7y)/ 18 (9.4y)/
			25 (9.2y) (28.0%)		25 (10.0y) (28.0%)
E	Broodmare	30 Jun, 2002	6 (12.7y)/ 23 (10.7y)/		
			29 (11.1y) (20.7%)		
F	Broodmare	30 Jun, 2002	3 (8.7y)/ 9 (9.7y)/		
			12 (9.4y) (25.0%)		
G	Broodmare			11 Mar, 2015	5 (14.0y)/ 25 (11.0y)/
					30 (11.5y) (16.7%)
H	Broodmare			17 Jan, 2014	5 (11.4y)/ 34 (10.8y)/
					39 (10.9y) (12.8%)
I	Broodmare			28 Jan, 2016	3 (11.3y)/ 19 (9.3y)/
					22 (9.6y) (13.6%)
J	Broodmare			31 Oct, 2016	2 (11.5y)/ 9 (12.2y)/
					11 (12.1y) (18.2%)
Subtotal	Stallion		3 (8.0y)/ 25 (9.6y)		20 (13.3y)/ 39 (10.1y)/
			28 (9.1y) (10.7%)		59 (11.2y) (33.9%)
	Broodmare		34 (10.3y)/ 104 (9.6y)		60 (12.0y)/174 (10.2y)/
			138 (9.8y) (24.6%)		234 (10.7y) (25.6%)
Total			37/166 ^4^ (22.3%)		80/293 (27.3%)

^1^ Number in parenthesis is the average age (years) of antibody-positive horses; ^2^ Number in parenthesis is the average age of tested horses; ^3^ Number in parenthesis is antibody-positive rate; ^4^ Positive sera/ tested sera.

**Table 6:** Relationship between average ages of EFV antibody-positive horses and EFV antibody-positive rates.

Age (years)	Farm A		Farm C	
	2002	2015	2002	2015
	Positive/Tested	Positive/Tested	Positive/Tested	Positive/Tested
4	1/2 (50.0%) ^1^	1/1 (100.0%)	0/2	
5	0/2		0/7	0/3
6	0/1	0/7	2/7 (28.6%)	1/5 (20.0%)
7	0/5	2/5 (40.0%)	4/9 (44.4%)	2/11 (18.2%)
8	0/3	2/6 (33.3%)	1/4 (25.0%)	3/13 (23.1%)
9	0/4	1/3 (33.3%)	2/5 (40.0%)	2/9 (22.2%)
10	2/3 (66.7%)	1/3 (33.3%)	2/10 (20.0%)	6/19 (31.6%)
11	0/2	0/1	2/11 (18.2%)	4/10 (40.0%)
12	0/1	0/1	3/6 (50.0%)	5/9 (55.6%)
13	0/1	1/2 (50.0%)	1/2 (50.0%)	2/4 (50.0%)
14	0/1	0/3	0/4	4/8 (50.0%)
15	0/1	2/2 (100.0%)	1/2 (50.0%)	3/7 (42.9%)
16	0/1	3/3 (100.0%)	0/1	1/2 (50.0%)
17		2/3 ^3^ (66.7%)	0/2	4/5 ^6^ (80.0%)
18				1/1 ^6^ (100.0%)
19				0/1 ^6^
20				
21				
22		1/1 ^4^ (100.0%)		
23	0/1	2/2 ^5^ (100.0%)		
24		0/1 ^6^		
Subtotal 4y to 9y	1/17 (5.9%)	6/22 (27.3%)	9/34 (26.5%)	8/41 (19.5%)
10y to 14y	2/8 (25.0%)	2/10 (20.0%)	8/33 (24.2%)	21/50 (42.0%)
15y to 24y	0/3	10/12 (83.3%)	1/5 (20.0%)	9/16 (56.3%)
total	3/28 (10.7%)	18/44 (40.9%)	18/72 (25.0%)	38/107 (35.5%)
Average age	8.0y/ 9.6y/ 9.1y ^2^	13.7y/ 9.7y/ 11.3y	9.6y/ 9.4y/ 9.4y	12.0y/ 9.9y/ 10.6y

^1^ Number in parenthesis is antibody-positive rate; ^2^ Average age of antibody-positive horses/ average age of antibody-negative horses/ average age of total tested horses; ^3^ One positive horse also had the antibody in 2002 (4-year-old horse). The remaining two horses were not reared in 2002; ^4^ This horse was reared in 2002 (9 years old) and was antibody-negative in 2002; ^5^ Both horses were reared in 2002 (10 years old) and one horse was antibody-positive in 2002; ^6^ These horses were not reared in 2002.
